# A Comparison of *MGMT* Testing by MSP and qMSP in Paired Snap-Frozen and Formalin-Fixed Paraffin-Embedded Gliomas

**DOI:** 10.3390/diagnostics13030360

**Published:** 2023-01-18

**Authors:** Milica Lazarević, Nikola Jovanović, Vladimir J. Cvetković, Svetlana Tošić, Jelena Vitorović, Slaviša Stamenković, Vesna Nikolov, Nataša Vidović, Jelena Kostić Perić, Marija Jovanović, Tatjana Mitrović

**Affiliations:** 1 Laboratory for Molecular Biology and Biotechnology, Department of Biology and Ecology, Faculty of Sciences and Mathematics, University of Niš, 18000 Niš, Serbia; 2 Faculty of Medicine, Clinic of Neurosurgery, Clinical Centre, University of Niš, 18000 Niš, Serbia; 3 Faculty of Medicine, Pathology and Pathological Anathomy Centre, Clinical Centre, University of Niš, 18000 Niš, Serbia; 4 Laboratory for Molecular Biomedicine, Institute of Molecular Genetics and Genetic Engineering, University of Belgrade, 11042 Belgrade, Serbia

**Keywords:** MSP, qMSP, glioma, *MGMT*, FFPE, prognostic factor

## Abstract

Comparative analysis of the conventional methylation-specific PCR (MSP) vs. the quantitative MSP (qMSP) assessment of the O^6^-methylguanine-DNA methyltransferase (*MGMT*) promoter methylation status in 34 snap-frozen (SF) glioma samples was performed. The accuracy of the semi-quantitative MSP was compared with the corresponding qMSP semi-quantitative values using two semi-quantitative cut-off values (0—unmethylated and 1—weakly methylated) to discriminate methylated from unmethylated samples. In the case of the cut-off value 0, MSP test showed 80.0% sensitivity and 78.9% specificity compared to the reference qMSP analysis. However, when using the cut-off value 1, the diagnostic accuracy of the MSP test was significantly higher (85.7% sensitivity, 85.2% specificity). Fleiss’ Kappa statistical analyses indicated moderate agreement (Fleiss’ Kappa Coefficient = 0.509; 70.59% agreement) between MSP and qMSP semi-quantitative measurements of *MGMT* promoter methylation in glioma patients, justifying the conventional MSP use in diagnostics and confirming its high reliability. Further, we aimed to compare the validity of SF and formalin-fixed paraffin-embedded (FFPE) glioma samples for *MGMT* testing. Statistical analyses indicated moderate overall agreement of FFPE glioma samples and SF MSP semi-quantitative measurements (Fleiss’ Kappa Coefficient = 0.516/0.509; 70.0% agreement) and emphasized their low reliability in the assessment of highly methylated *MGMT* promoter samples.

## 1. Introduction

According to Global Cancer Statistics (GLOBOCAN 2020), 308,102 new cases and 251,329 deaths of brain and central nervous system (CNS) cancers are registered worldwide (1.6% and 2.5% of all cancers, respectively) [1]. The burden of brain and CNS cancers incidence and mortality is rapidly growing, affecting healthcare systems and quality of life [2]. The highest incidence of brain and CNS cancers is observed in Western Europe, Central Europe and North America, while the highest mortality is determined in Central Europe, Tropical Latin America and Australasia [2]. Serbia is identified as the fifth leading country by age-standardized mortality rate (ASMR) (6.85 per 100, 000 population) of brain and CNS cancers among the 204 countries [2].

Gliomas represent the most common form of CNS neoplasms that originate from glial cells and include 22.4% of all primary brain and CNS cancers and 78.2% of all malignant brain and CNS cancers [3]. The vast majority of gliomas belong to diffuse gliomas which are highly infiltrative, frequently recurrent and mostly incurable malignancies. Based on recommendations by the Consortium to Inform Molecular and Practical Approaches to CNS Tumor Taxonomy—Not Official World Health Organization (WHO) (cIMPACT-NOW) WHO created the latest classification of CNS tumors (5th edition, 2021) which includes following adult-type diffuse gliomas: astrocytoma, isocitrate dehydrogenases (*IDH*)-mutant; oligodendroglioma, *IDH*-mutant and 1p/19q-codeleted; and glioblastoma, *IDH*-wildtype [4].

The tumor suppressor gene for O^6^-methylguanine-DNA methyltransferase (*MGMT*), located on chromosome 10q26.3, encodes a highly evolutionarily conserved enzyme involved in DNA repair [5,6,7,8,9,10]. This enzyme catalyzes the methyl group transfer from O^6^-methylguanine in DNA to one of its cysteine residues and eliminates damage from alkylating agents [5]. Transcriptional silencing of *MGMT* gene and increased tumor sensitivity to alkylating drugs was observed in gliomas, colorectal carcinomas, ovarian carcinomas, non-small cell lung carcinomas, head and neck carcinomas, lymphomas, etc. [11,12]. Esteller and colleagues detected aberrant *MGMT* promoter methylation in 40% of the gliomas and defined it as a decisive, independent, prognostic factor associated with regression of the tumor and overall survival in patients treated with radiotherapy and alkylating agent bis-chloroethyl nitrosourea (BCNU) [12,13]. Later, Hegi et al. underlined the predictive value of the *MGMT* gene silencing for patients receiving radiotherapy and adjuvant alkylating agent temozolomide (TMZ) [14,15].

Nowadays, it is generally accepted that the loss of *MGMT* expression is primarily a consequence of epigenetic modification of the *MGMT* promoter (i.e., hypermethylation of 98 CpG sites clustered in CpG island (CGI), which spans 762 bp (from −452 bp to +308 bp of the first exon of the *MGMT* gene)) [16,17,18,19]. Malley and associates identified two distinct regions within *MGMT* CGI, termed differentially methylated regions 1 and 2 (DMR1 and 2), where methylation status is strongly correlated with *MGMT* expression [17]. This study indicated DMR2 as critical for the transcriptional control of *MGMT* and the optimal target for methylation testing [17].

For over two decades, alkylating agents have been the best chemotherapeutic agents for glioma treatment [13,14,15,20,21,22,23]. The treatment efficacy depends on the methylation status of the *MGMT* promoter in tumor tissue, meaning that only those with methylated promoters will significantly respond and have prolonged survival. These findings contributed to *MGMT* promoter methylation’s prognostic and predictive importance in gliomas and led to the European Association of Neuro-Oncology (EANO) recommendation that *MGMT* testing in gliomas should be included in the treatment decision process [24].

A variety of methods and platforms have been developed to detect *MGMT* promoter methylation in gliomas, including methylation-specific PCR (MSP), quantitative methylation-specific PCR (qMSP), multiplex ligation-dependent probe amplification (MLPA), pyrosequencing (PSQ), methylation-sensitive high-resolution melting (MS-HRM), Next Generation Sequencing (NGS) and immunohistochemistry (IHC) [25]. 

The most widely used method for detection of promoter methylation, MSP, was established by Herman et al. in 1996 [12,13,25,26]. Here, standard MSP primers, which determine the methylated or unmethylated status of *MGMT* promoter semi-quantitatively, bind to +115 to +137 (forward) and +174 to +195 (reverse) within the previously described DMR2 region of *MGMT* promoter. Later, a novel quantitative version of MSP (qMSP) using the same primers is developed [27,28].

Although *MGMT* testing is clinically routine in many countries, there are still many controversies concerning methods and cutoff levels [25]. Moreover, there are difficulties with obtaining snap-frozen samples (SF) from operating rooms for rare cancers, such as gliomas, because histologic diagnosis is prioritized to SF storage, which reduces the collection of SF samples for precise molecular analysis. Thus, researchers often reach out for archived formalin-fixed paraffin-embedded (FFPE) samples. Furthermore, it should be emphasized that FFPE tissues represent the most extensive available collection of gliomas (and other tumor and biological specimens) suitable for decades of storage in pathological archives worldwide. Therefore, optimization of methods for *MGMT* testing from FFPE is needed for future molecular studies.

There two major goals in our study: the first, to compare two different methods for *MGMT* methylation status assessment (MSP vs. qMSP), and the second, to compare the validity of the snap-frozen (SF) and the formalin-fixed paraffin-embedded (FFPE) glioma samples as sources of DNA for previous testing methods.

## 2. Materials and Methods

### 2.1. Tumor Specimens

The samples of 34 patients diagnosed with glioma at the Neurosurgery Clinic at the Clinical Centre of Niš were analyzed in this study. Tumor specimens were acquired from patients who underwent surgery from June 2013 till December 2019 at the Neurosurgery Clinic, University of Niš, Serbia with written inform consent and approval from the Ethics Committee from the Clinical Centre and Faculty of Medicine (permission No. 01-2113-10, 1 April 2013). SF glioma samples were snap frozen and collected in RNAlater^®^ (Qiagen, Hilden, Germany) and stored at −80 °C and FFPE glioma samples were stored at the dark and dry place at room temperature prior the genomic DNA isolation.

### 2.2. Genomic DNA Isolation and Bisulfite Conversion

Extraction of genomic DNA was conducted utilizing the QIAamp^®^ DNA Mini Kit (Qiagen, Hilden, Germany) from 25 mg of SF sample and QiAamp DNA FFPE Tissue Kit (Qiagen, Hilden, Germany, Catalogue No. 56404) from eight freshly cut sections with a thickness of 10 µm from FFPE sample. The genomic DNA (2 µg) was modified by sodium bisulfite using EpiTect^®^ Bisulfite Kit (Qiagen, Hilden, Germany) for the SF DNA sample and Epitect Plus FFPE bisulfite kit (Qiagen, Hilden, Germany, Catalogue No. 59144) for FFPE DNA sample. BioSpec–nano UV–Vis Spectrophotometer (Shimadzu, Kyoto, Japan) was utilized for the determination of quantity and quality of isolated DNA and bisulfite-converted samples. The DNA samples were quality checked by agarose gel electrophoresis (2% agarose gel).

### 2.3. Methylation-Specific Polymerase Chain Reaction (MSP) 

#### 2.3.1. MSP Analysis of the SF Samples

After the optimization of MSP reactions considering similar studies, the conventional MSP analysis was carried out in a total volume of 20 μL containing 1× PCR buffer with 1.5 mM MgCl_2_ (Qiagen Hilden, Germany), 10 pM of appropriate forward and reverse primer, 0.2 μM dNTP mix, 1U HotStar Taq polymerase (Qiagen Hilden, Germany) and 125 ng of bisulfite-converted template DNA (Table 1) [13,27,28].

MSP amplification was conducted in Mastercycler Gradient (Eppendorf, Hamburg, Germany) utilizing following programme: 95 °C for 15 min, then 35 cycles of 95 °C for 50 s, 59 °C for 50 s and 72 °C for 50 s and final extension at 72 °C for 10 min. Control PCR reactions were carried out using EpiTect PCR Control DNA set (Qiagen Hilden, Germany), which consisted of unconverted unmethylated human DNA, unmethylated bisulfite converted human DNA and methylated bisulfite converted human DNA as DNA templates. Non-template control PCR reactions were included, and reactions were conducted in duplicate.

MSP products were visualized by UV light on a 2% agarose gel containing ethidium bromide. 

For qualitative *MGMT* promoter methylation analysis, a visible band of methylated *MGMT* promoter product indicated a positive *MGMT* methylation status, while the absence of such PCR product was considered as a negative methylation status of *MGMT* [28]. 

For the semi-quantitative MSP analysis, gel images were subject to ImageJ software analysis (National Institute of Health, Bethesda, Rockville, MD, USA; https://imagej.nih.gov/ij/, accessed on 20 November 2021) for calculation of the ratio of the methylated and unmethylated *MGMT* promoter product’s fluorescence intensities. Following the ImageJ analysis, one of three semi-quantitative values (0—unmethylated; 1—weakly methylated; 2—strongly methylated) were assigned to each sample.

#### 2.3.2. MSP Analysis of the FFPE Samples

Similarly to the SF MSP analysis, the MSP reactions for the FFPE samples were run in a total volume of 20 µL containing 0.2 μM dNTP mix, 1× PCR buffer with 1.5 mM MgCl_2_ (Qiagen, Hilden, Germany), 10 pM of appropriate forward and reverse primer (Table 1) and 125 ng of bisulfite-converted template DNA. Amplification reactions were performed in a Mastercycler Gradient (Eppendorf, Hamburg, Germany) using the previously mentioned MSP program: 95 °C for 15 min, then 35 cycles of 95 °C for 50 s, 59 °C for 50 s, and 72 °C for 50 s, and final extension at 72 °C for 10 min. However, these MSP reactions were conducted with higher amounts of HotStar Taq polymerase (4U) according to suggestions from the previous study of our team [29]. MSP gel images were subjected to ImageJ software analysis (National Institute of Health, Bethesda, Rockville, MD, USA) to measure the fluorescence intensity of methylated and unmethylated *MGMT* promoter bands. Intensity ratio values were assessed by ImageJ analysis, and semi-quantitative *MGMT* promoter methylation values were calculated for glioma samples.

#### 2.3.3. qMSP Analysis of the SF Samples

The AriaMx qPCR machine (Agilent Technologies, Santa Clara, CA, USA) was utilized for the qMSP analyses of the *MGMT* promoter methylation status. QuantiNova SYBR^®^ Green PCR kit (Qiagen, Hilden, Germany) was used in the modified amplification protocol presented by Håvik and colleagues [28]. A 20 µL reaction mixture included: 1× QuantiNova Sybr^®^ Green master mix, 1× QN ROX^TM^ reference dye, 10 pM of appropriate forward and reverse primer, and 125 ng of bisulfite-converted template DNA. The program for the qMSP amplification was following: 95 °C for 2 min, then 35 cycles of 95 °C for 5 s, and 60 °C for 11 s. All qMSP reactions carried out in duplicate. Quantitative methylation levels of the samples were estimated relative to methylated and bisulfite-converted control DNA (Qiagen, Germany) using the 2^−∆∆Ct^ quantification approach. Additionally to the *MGMT* promoter primer sets, control *ALU–C4* primers were used for normalization: forward 5’-GGTTAGGTATAGTGGTTTATATTTGTAATTTTAGTA-3’ and reverse 5’-ATTAACTAAACTAATCTTAAACTCCTAACCTCA-3’ [30]. The amplicon size with *ALU C4* primers was 98 bp.

The PCR efficiency (E) was determined for the methylated *MGMT* promoter and *ALU C4* amplification. qMSP reactions were conducted with serial dilutions (factor-2× for the *MGMT* promoter product and factor-10× for the *ALU C4* product), a standard curve was generated, and its slope (which defines the E value) was calculated in Aria MxPro—Mx3005P software.

The quantitative level of the *MGMT* promoter methylation was expressed as the Percentage of Methylated Reference (PMR). PMR value was assessed by dividing the methylated *MGMT/ALU C4* relative quantity ratio in a sample and the methylated *MGMT/ALU C4* relative quantity ratio in fully methylated human genomic DNA control and multiplying by 100 [28]. A threshold value for scoring methylation-positive samples was defined based on the qMSP result in meningiomas, which had PMR values of zero [28]. For statistical comparison with the conventional MSP evaluation, quantitative values were converted to semi-quantitative scores (unmethylated, weakly methylated, and strongly methylated). Samples with 0% PMR were assigned to the unmethylated group, the ones with PMR ranging from 0–100% were assigned to the weakly methylated group, and samples with the PMR values higher than 100% were evaluated as strongly methylated.

### 2.4. Statictical Analyses

Statistical analyses were performed using the SPSS 15.0 software package (IBM Corp., Armonk, NY, USA) with *p* < 0.05 considered significant. Cross-tabulation analysis (contingency table analysis) was used for the comparison of the categorical (semi-quantitative) measurements of the MGMT promoter methylation assessed with MSP and the reference qMSP assay. To compare the three variants of semi-quantitative assessment of MGMT methylation status, Fleiss’ Kappa test was also conducted.

## 3. Results

### 3.1. Optimization of the MGMT Promoter MSP Reaction Conditions 

#### 3.1.1. Semi-Quantitative MSP Analysis of SF Samples

**Figure 1:**
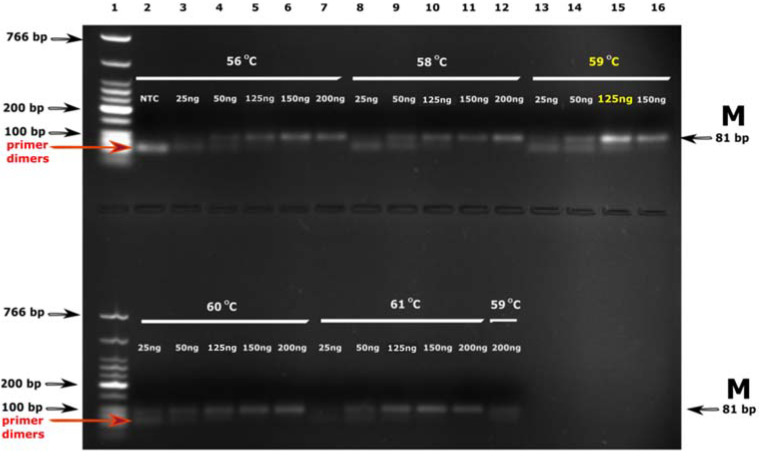
Optimization of the MSP amplification from SF samples by variation of the annealing temperature (°C) and the amount of bisulfite-DNA template (ng). 1—DNA marker (Low Molecular Weight DNA ladder, New England BioLabs, Ipswich, MA, USA); 2–16—MSP reactions for the methylated *MGMT* promoter product (M); NTC—non-template control.

**Figure 2:**
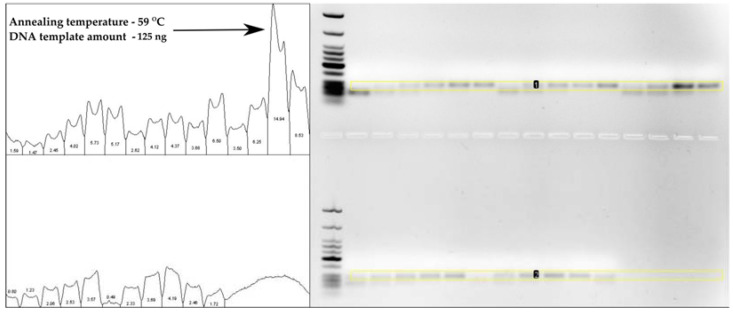
ImageJ software analysis of the methylated *MGMT* promoter MSP amplification conditions.

The optimization of the MSP reaction mixture for semi-quantitative evaluation of the *MGMT* promoter methylation status was performed considering previously recommended reaction conditions [13,27,28]. Firstly, sets of MSP reactions were designed to choose the adequate *MGMT* primers annealing temperature and the amount of template DNA. MSP (PCR) products were analyzed by agarose gel electrophoresis, and the relative DNA yield/quality was calculated by the ImageJ software. After such optimization, the annealing temperature of 59 °C and the amount of bisulfite-converted DNA of 125 ng were chosen (Figure 1 and Figure 2).

**Figure 3:**
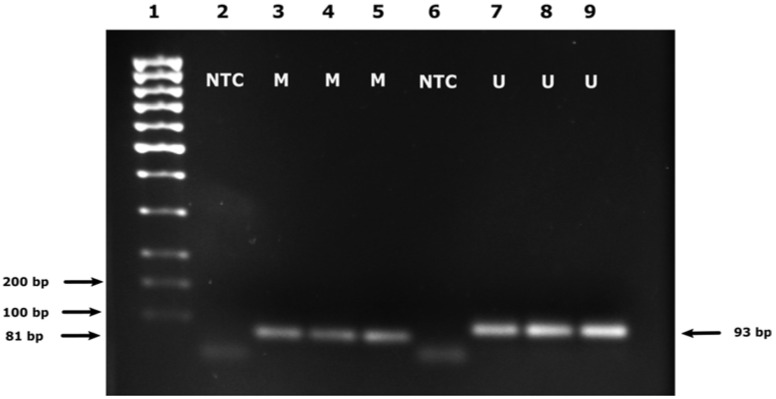
Pilot MSP amplification of unmethylated and methylated *MGMT* products was performed for a single SF glioblastoma sample in triplicate. 1—DNA marker (Low Molecular Weight DNA ladder, New England BioLabs, Ipswich, MA, USA); 2—NTC—non-template control (primer set for methylated *MGMT*); 3–5—Methylated *MGMT* promoter MSP products (M); 6—NTC—non-template control (primer set for unmethylated *MGMT*); 7–9—Unmethylated *MGMT* promoter MSP products (U).

Following such optimization, the pilot *MGMT* promoter MSP test for single SF sample confirmed the adequacy of chosen reaction conditions for both unmethylated and methylated sets of primers (Figure 3).

After confirmation of optimal MSP reaction’s conditions, the mass MSP analysis was conducted. Each SF sample was analyzed in duplicate, and mean fluorescence was calculated for each sample from two appropriate (methylated/unmethylated) bands on a gel. The results of the SF MSP semi-quantitative evaluation of 34 glioma samples are presented conjointly with the rest of the results in Table 2.

#### 3.1.2. Semi-Quantitative MSP Analysis of FFPE Samples

**Figure 4:**
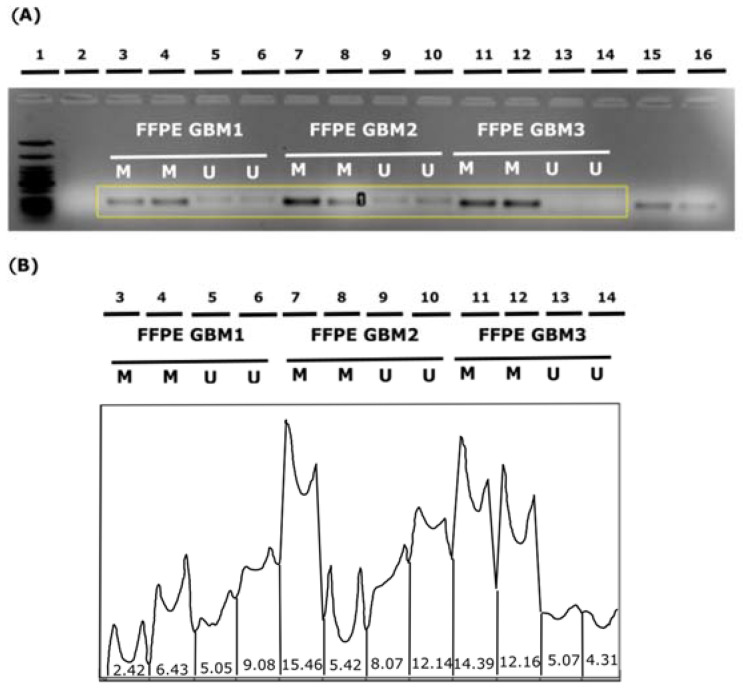
An example of ImageJ software analysis performed after the MSP amplification of *MGMT* promoter in FFPE glioblastoma (GBM) samples. (**A**) Agarose gel image prepared for the ImageJ analysis (the yellow box represents the ImageJ analysis area): 1—DNA marker (Low Molecular Weight DNA ladder, New England BioLabs, Ipswich, USA); 2—NTC—non-template control (primer set for methylated *MGMT*); 3–14—Methylated *MGMT* promoter MSP products (M) alongside corresponding Unmethylated MGMT promoter MSP products (U) of three GBM samples. (**B**) Histogram chart representing relative fluorescence intensity of individual bands of MSP products on the gel.

Semi-quantitative MSP assay with FFPE samples was performed considering the previous findings of our research team [29]. Thus, in comparison with SF samples analysis, MSP reactions were additionally optimized by increasing the amount of HotStar Taq polymerase to 4U [29]. Similarly to SF MSP analysis, gel electrophoresis images were submitted to the ImageJ software analysis (Figure 4). 

The MSP semi-quantitative results of 20 FFPE samples are presented alongside the corresponding SF MSP and SF qMSP analyses in Table 2.

#### 3.1.3. Semi-Quantitative qMSP Analysis of FF Samples

**Figure 5:**
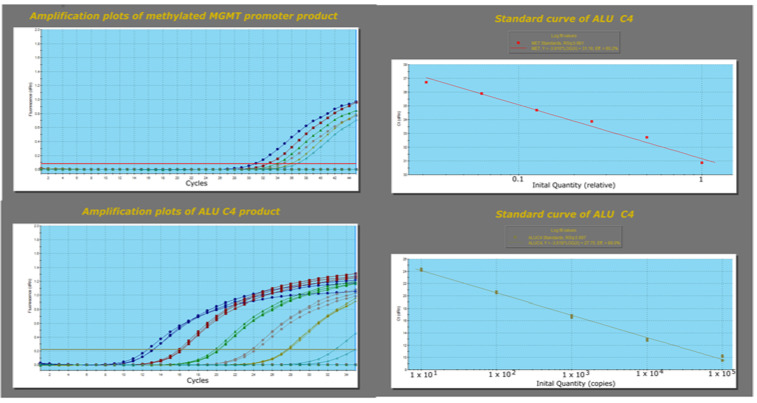
Amplification plots for methylated *MGMT* promoter qMSP product and *ALU C4* normalizer qMSP product.

The level of *MGMT* promoter methylation was initially evaluated by qMSP as the percentage of methylated reference (PMR) using the previously reported protocol [28]. Before the mass qMSP evaluation, the efficiencies of *MGMT* promoter and *ALU C4* qMSP amplification were determined on a randomly chosen GBM sample. After making serial dilutions of the template DNA and generating the linear regression curves, the calculated efficiencies were 80.2% for the methylated *MGMT* promoter amplification and 89% for the *ALU C4* normalizing assay. These values were entered in Aria MxPro—Mx3005P software during calculating the PMR values of all analyzed samples (Figure 5). 

**Figure 6:**
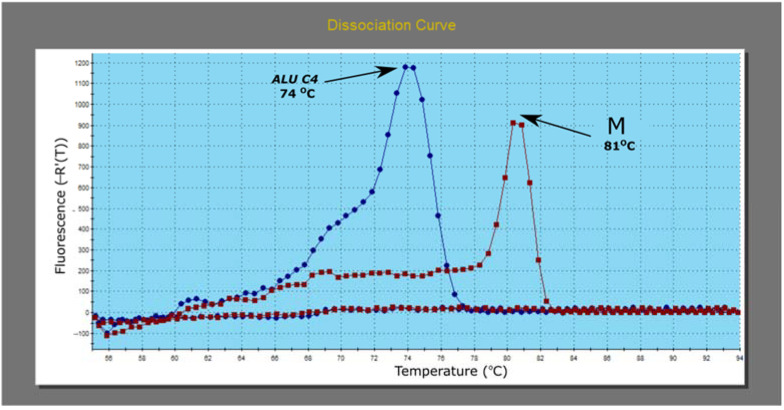
The Dissociation Curve analysis following test qMSP amplification of methylated *MGMT (M)* and *ALU C4* normalizing assay.

The Dissociation Curve analysis confirmed the presence of the specific qMSP products (*ALU C4* and methylated *MGMT)* and the absence of nonspecific products (Figure 6).

**Figure 7:**
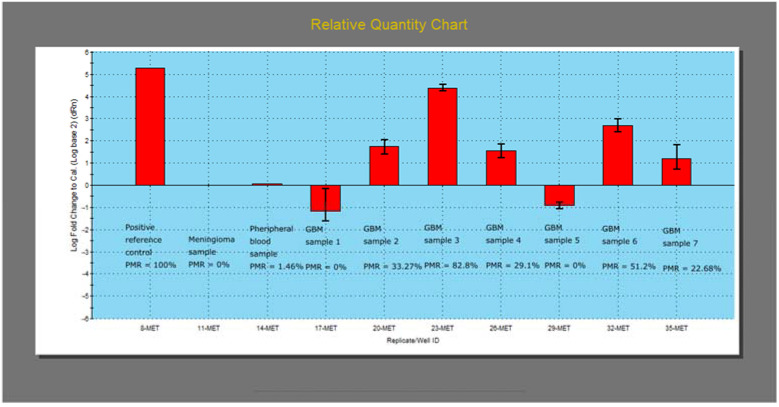
Relative Quantity Chart for methylated *MGMT* promoter qMSP product with the PMR values calculated for each sample.

After the optimization reactions, the mass qMSP evaluation was conducted. PMR values were calculated for each glioma sample (N = 34) and appropriate positive and negative controls (reference positive control, meningioma, peripheral blood) (Figure 7).

The calculated PMR values are later converted to unmethylated (0% PMR), weakly methylated (0–100% PMR), and strongly methylated (>100% PMR) semi-quantitative values of *MGMT* promoter methylation. Such values are presented in Table 2, conjointly with the other MSP results.

### 3.2. Comparison of Three Different MSP Assays in MGMT Promoter Methylation Evaluation 

**Figure 8:**
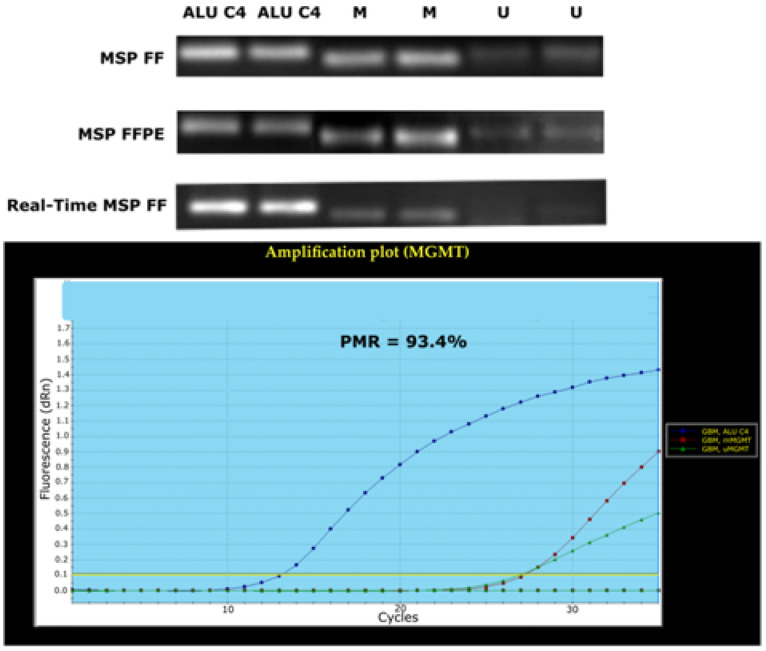
Juxtaposing MSP products obtained by three MSP approaches in single patient *MGMT* promoter methylation analysis: conventional MSP analysis of SF sample, conventional MSP analysis of FFPE sample, and qMSP (Real-Time MSP) analysis of SF sample.

The agarose gel electrophoresis analysis of MSP products for corresponding samples (SF and FFPE) originating from the same GBM patient is presented in Figure 8.

Qualitative evaluation (presence of the methylated *MGMT* product on a gel) was consistent for 16/20 (80%) patients for whom all three MSP assay approaches were available. Samples of two patients (10%) were positively evaluated by qMSP assay and negatively by the SF MSP and FFPE MSP, one patient was evaluated positively solely by the FFPE MSP analysis (5%), and one patient evaluated positively by the SF and FFPE MSP, but not qMSP (5%). 

#### 3.2.1. Comparison of Semi-Quantitative MSP and Semi-Quantitative qMSP Analysis of SF Glioma Samples

For statistical comparison of the conventional MSP and qMSP results, the quantitative MSP data (PMR) was brought down to semi-quantitative values of 0—unmethylated, 1—weakly methylated, and 2—strongly methylated *MGMT* promoter, as mentioned in Materials and Methods (Table 2). 

The accuracy of the semi-quantitative MSP assay was tested by comparison with the corresponding qMSP semi-quantitative values obtained for 34 patients (Table 3). Firstly, the 0 semi-quantitative cut-off value was used to discriminate positively methylated from unmethylated samples. With a cut-off value of 0, conventional semi-quantitative MSP test results showed the following properties when compared to the reference qMSP analysis:

- True positive rate (sensitivity)—80.0%;
- False negative rate (Type 2 error)—20.0%;
- False positive rate (Type 1 error)—21.1%;
- True negative rate (specificity)—78.9%.

When using a cut-off value of 1, conventional semi-quantitative MSP test results showed the following properties in comparison with the reference qMSP analysis (Table 4):

- True positive rate (sensitivity)—85.7%;
- False negative rate (Type 2 error)—14.3%;
- False positive rate (Type 1 error)—14.8%;
- True negative rate (specificity)—85.2%

Statistical analyses of coincidence in semi-quantitative measurements between MSP and qMSP indicated moderate agreement (Fleiss’ Kappa Coefficient = 0.509; 70.59% agreement) (Table 5).

#### 3.2.2. Comparison of MSP Analysis of FFPE and SF Glioma Samples

Fleiss’ Kappa statistical analyses indicated slightly higher agreement between FFPE MSP and SF MSP semi-quantitative measurements in comparison with the agreement between FFPE and SF qMSP measurements. The level of coincidence of the semi-quantitative measurements obtained for FFPE and SF samples are presented in Table 6 and Table 7.

## 4. Discussion

The recent international survey of the most common methods utilized for the evaluation of the *MGMT* promoter methylation status, the most valuable prognostic factor of glioma, emphasized the MSP as the first method of choice among diagnostics laboratories due to its good simplicity, reproducibility, cost-effectiveness, and best correlation with clinical outcome [25]. However, this survey also revealed the significant variation within definitions of cut-off values, which are, for the central part of the 152 respondents, being internally defined at their pathology departments or the associated companies performing the testing. Some of the respondent laboratories noted the advantage of the subdivision of the results of *MGMT* promoter methylation into more than two groups, such as “unmethylated”, “weakly methylated”, and “strongly methylated”. This approach could be used to circumvent the “gray zone” diagnostic uncertainty of MSP testing, which is repeatedly documented in the literature [31,32]. 

It was reported that simple qualitative MSP-based methods are likely inferior to quantitative methods and that the dichotomous classification of methylated versus unmethylated may be the oversimplified approach of *MGMT* promoter evaluation [32,33]. Given that, our research team conducted several studies concerning the prognostic significance of semi-quantitative *MGMT* promoter methylation status obtained with both conventional MSP and Real-Time MSP assays among the Serbian population of glioblastoma/diffuse glioma patients [34,35,36]. We also made efforts to confront the issues regarding the conventional MSP test performed on the FFPE samples and proposed the optimum reaction conditions for such testing [29]. 

Although the conventional qualitative MSP analysis may be more affordable and prevalent (which is particularly important in European countries outside of the EU), qMSP is a more standardized, high-throughput method that brings about more reliable results and is considered more suitable for use in routine testing [28]. In the present study, we intended to explore whether the semi-quantitative approach of the conventional MSP method (initially presented by Christians and colleagues) could bring results as nearly reliable as the qMSP assay [27]. The reliability of such an MSP approach was especially tested in the case of using the FFPE samples, which would enable more widespread testing of the *MGMT* promoter methylation among diagnostic laboratories.

Thus, we tested the diagnostic accuracy of semi-quantitative MSP assays conveyed on 34 SF and 20 FFPE glioma samples for which the correspondent qMSP results were available. The threshold value of the reference qMSP method was initially set using the benign meningioma control sample, according to the recommendation from the literature [28]. Later, the obtained PMR qMSP results were translated to semi-quantitative values and statistically compared to their semi-quantitative MSP counterparts.

In addition to the reference study recommendations and to ensure the highest possible reliability of MSP assay performed on SF samples, sets of optimization reactions were conducted to detect the most suitable annealing temperature and the amount of the bisulfite-converted template DNA (Figure 1). Before the mass MSP testing, its validity was confirmed for the single GBM sample conducted in triplicate (Figure 3). The semi-quantitative MSP values of *MGMT* methylation were obtained using the ImageJ software according to the previously described protocol [27]. The MSP assay concerning the FFPE samples was optimized according to our previous findings to ensure its validity [29]. The qMSP efficiency calculation of the methylated *MGMT* product and *ALU C4* normalizing assay proved the satisfactory qMSP amplification conditions.

The results obtained in the present study suggest the high reliability of the semi-quantitative MSP assay performed on SF samples. Compared to the reference qMSP assay, it showed good accuracy (sensitivity 80.0% and specificity 78.9%) in evaluating the samples with positively methylated *MGMT* promoter (weakly and strong methylation) and unmethylated *MGMT.* However, when the cut-off value for differentiating methylated from unmethylated samples was set to 1, that accuracy was substantially higher (sensitivity 85.7% and specificity 85.2%). Such an observation could be explained by the existence of the mentioned “gray zone” of the MSP diagnostic uncertainty, which is possibly “settled” within the “weakly methylated *MGMT* promoter” range of the results [31]. Thus, concerning the interpretation of the semi-quantitative results obtained with the conventional MSP, in the case of the “weakly methylated” result, we would suggest the use of complementary *MGMT* promoter methylation evaluation assay (e.g., immunohistochemistry). The accuracy of the semi-quantitative MSP assay was further confirmed by the calculation of the level of coincidence in semi-quantitative measurements with the reference qMSP results, which showed moderate overall agreement (Fleiss’ Kappa Coefficient = 0.509; 70.59% agreement). However, the agreement analysis of the individual semi-quantitative scores of the methylation level (0,1,2) confirmed the weakness of the conventional *MSP* method in the assessment of weakly methylated samples (Fleiss’ Kappa Coefficient = 0.28; *p* value—0.51). In contrast, MSP showed substantial agreement with the reference qMSP method in recognizing unmethylated and hypermethylated *MGMT* promoter samples.

Fleiss’ Kappa statistical analyses indicated moderate overall reliability of MSP assay conveyed on the FFPE samples when compared to the MSP of SF samples (Fleiss’ Kappa Coefficient = 0.516; 70.0% agreement). Such comparison with the reference qMSP results indicated its moderate overall reliability as well (Fleiss’ Kappa Coefficient = 0.509; 70.0% agreement). Although these results confirm the substantial agreement in detection of unmethylated samples, the agreement in assessing weakly methylated samples was only fair to moderate. Concerning the detection of highly methylated samples, only a slight to fair agreement was found. This could be explained by the fact that the use of FFPE induces non-reproducible bisulfite conversion leading to unreliable and inconsistent results for methylation levels [37,38]. Poor DNA quality of FFPE isolates is mainly caused by formalin fixation, which induces the formation of DNA-protein crosslinks that are difficult to remove by lysis protocols [37,38]. Based on the present findings, our conclusion would be that the semi-quantitative MSP approach performed on FFPE samples brings about acceptable results, although the qMSP method should have priority in their analyses.

## 5. Conclusions

Comparative analysis of two methods for the determination of *MGMT* marker in gliomas, MSP and qMSP, is performed. The sensitivity and specificity of MSP in SF samples compared to reference qMSP were 85.7% and 85.2%, respectively, and depended on the chosen cutoff value for differentiating methylated from unmethylated glioma samples. Concerning the utilization of archived FFPE glioma samples for *MGMT* testing, the semi-quantitative MSP approach performed on FFPE samples brings about acceptable results. However, the qMSP method should have priority in their analyses. Suspicious, “weakly methylated” samples should be submitted to additional IHC analysis to provide better sensitivity and specificity for determining the *MGMT* status.

## Figures and Tables

**Table 1:** *MGMT* primer sequences utilized in MSP analyses.

Gene	Primer Sequence (5′-3′)	Amplicon Size (bp)	Reference
Unmethylated *MGMT* promoter (U)	F: TTTGTGTTTTGATGTTTGTAGGTTTTTGT R:AACTCCACACTCTTCCAAAAACAAAACA	93	[13]
Methylated *MGMT* promoter (M))	F: TTTCGACGTTCGTAGGTTTTCGC R: GCACTCTTCCGAAAACGAAACG	81	[13]

**Table 2:** List of analyzed samples and *MGMT* promoter methylation status analysis.

Patient No	Diagnosis	IDH Mutation Status	WHO Grade	Conventional MSP (Semi-Quantitative) ^1^		qMSP (Quantitative) ^2^	qMSP (Semi-Quantitative) ^3^
				SF *MGMT* Methylation Status	FFPE *MGMT* Methylation Status	SF *MGMT* Methylation Status	SF *MGMT* Methylation Status
1.	GBM	*IDH1* wt ^4^	4	0	N/A	0	0
2.	GBM	*IDH1* wt	4	2	N/A	110.70	2
3.	GBM	*IDH1* wt	4	0	0	0	0
4.	GBM	*IDH1* wt	4	0	N/A	0	0
5.	GBM	*IDH1* wt	4	0	0	0	0
6.	GBM	*IDH1* wt	4	2	N/A	102.40	2
7.	GBM	*IDH1* wt	4	0	N/A	0	0
8.	GBM	*IDH1* wt	4	0	N/A	0	0
9.	GBM	*IDH1* wt	4	0	0	0	0
10.	rGBM ^5^	*IDH1* wt	4	1	1	33.27	1
11.	GBM	*IDH1* wt	4	1	1	29.10	1
12.	GBM	*IDH1* wt	4	0	0	0	0
13.	GBM	*IDH1* wt	4	0	0	0	0
14.	GBM	*IDH1* wt	4	0	0	0	0
15.	GBM	*IDH1* wt	4	2	1	103.40	2
16.	GBM	*IDH1* wt	4	1	2	108.08	2
17.	GBM	*IDH1* wt	4	0	0	31.95	1
18.	GBM	*IDH1* wt	4	0	N/A	0	0
19.	GBM	*IDH1* wt	4	2	1	145.20	2
20.	GBM	*IDH1* wt	4	0	0	82.80	1
21.	GBM	*IDH1* wt	4	0	1	0	0
22.	GBM	*IDH1* wt	4	2	2	51.20	1
23.	rGBM	*IDH1* wt	4	1	1	51.06	1
24.	rGBM	*IDH1* wt	4	2	1	9.30	1
25.	Astrocytoma	IDH-R132H ^6^	3	2	2	145.30	2
26.	GBM	*IDH1* wt	4	0	0	0	0
27.	GBM	*IDH1* wt	4	0	N/A	0	0
28.	Astrocytoma	IDH-R132H	3	2	N/A	0	0
29.	GBM	*IDH1* wt	4	2	2	0	0
30.	GBM	*IDH1* wt	4	0	N/A	0	0
31.	Astrocytoma	IDH-R132H	4	1	N/A	0	0
32.	GBM	*IDH1* wt	4	2	N/A	103.00	2
33.	GBM	*IDH1* wt	4	1	N/A	0	0
34.	GBM	*IDH1* wt	4	0	N/A	22.68	1

^1^ Semi-quantitative values of *MGMT* methylation status: 0—unmethylated *MGMT* promoter; 1—weak *MGMT* promoter methylation; 2—strong *MGMT* promoter methylation ^2^ Quantitative values of *MGMT* methylation status presented as the Percentage of Methylated Reference—PMR (%).; ^3^ Semi-quantitative values of *MGMT* methylation status based on quantitative MSP value (PMR): 0—unmethylated *MGMT* promoter (PMR = 0%); 1—weak *MGMT* promoter methylation (0% < PMR < 100%); 2—strong *MGMT* promoter methylation (PMR > 100%) ^4^ *IDH1* wt—isocitrate dehydrogenase 1 wild-type; ^5^ rGBM—recurrent glioblastoma; ^6^ *IDH1 R132*—isocitrate dehydrogenase 1 mutant.

**Table 3:** Sensitivity, specificity, and accuracy test of the conventional MSP semi-quantitative assay (for cut-off value 0).

Outcome of Semi-Quantitative MSP Test	*MGMT* Promoter Methylation Determined by qMSP		
	Positive	Negative	Raw Total
Positive	12 (80.0%)	4 (21.1%)	16 (47.1%)
Negative	3 (20.0%)	15 (78.9%)	18 (52.9%)
Column total	15 (44.11%)	19 (55.89%)	34 (100%)

**Table 4:** Sensitivity, specificity, and accuracy test of the conventional MSP semi-quantitative assay (for cut-off value 1).

Outcome of Semi-Quantitative MSP Test	*MGMT* Promoter Methylation Determined by qMSP		
	Positive	Negative	Raw Total
Positive	6 (85.7%)	4 (14.8%)	10 (29.4%)
Negative	1 (14.3%)	23 (85.2%)	24 (70.6%)
Column total	7 (20.58%)	27 (79.42%)	34 (100%)

**Table 5:** Fleiss’ Kappa statistical analyses of semi-quantitative MSP measurements and semi-quantitative qMSP measurements of corresponding 34 glioma samples.

No. Inspected	No. Matched	Percent	95% CI	
34	24	70.59	(52.52, 84.90)	
*MGMT promoter* *methylation score*	Kappa	SE Kappa	Z	*p* value (vs. >0)
0	0.585004	0.171499	3.41113	0.0003
1	0.280423	0.171499	1.63513	0.0510
2	0.607843	0.171499	3.54430	0.0002
Overall	0.509025	0.125059	4.07029	0.0000

**Table 6:** Fleiss’ Kappa statistical analyses of MSP measurements performed on FFPE samples and MSP measurements of corresponding 20 SF glioma samples.

No. Inspected	No. Matched	Percent	95% CI	
20	14	70.00	(45.72, 88.11)	
*MGMT promoter* *methylation score*	Kappa	SE Kappa	Z	*p* value (vs. >0)
0	1.00000	0.223607	4.47214	0.0000
1	0.28571	0.223607	1.27775	0.1007
2	0.06250	0.223607	0.27951	0.3899
Overall	0.51613	0.163055	3.16537	0.0008

**Table 7:** Fleiss’ Kappa statistical analyses of semi-quantitative MSP measurements (FFPE samples) and semi-quantitative qMSP measurements of 20 corresponding SF glioma samples.

No. Inspected	No. Matched	Percent	95% CI	
20	14	70.00	(45.72, 88.11)	
*MGMT promoter* *methylation score*	Kappa	SE Kappa	Z	*p* value (vs. >0)
0	0.699248	0.223607	3.12713	0.0009
1	0.466667	0.223607	2.08700	0.0184
2	0.215686	0.223607	0.96458	0.1674
Overall	0.509202	0.168957	3.01380	0.0013

## Data Availability

Data sharing not applicable. No new data were created or analyzed in this study. Data sharing is not applicable to this article.
